# Biologics in steroid resistant nephrotic syndrome in childhood: review and new hypothesis-driven treatment

**DOI:** 10.3389/fimmu.2023.1213203

**Published:** 2023-08-29

**Authors:** Andrea Angeletti, Maurizio Bruschi, Xhuliana Kajana, Edoardo La Porta, Sonia Spinelli, Gianluca Caridi, Francesca Lugani, Enrico Eugenio Verrina, Gian Marco Ghiggeri

**Affiliations:** ^1^ Division of Nephrology, Dialysis, Transplantation, IRCCS Istituto Giannina Gaslini, Genova, Italy; ^2^ Laboratory of Molecular Nephrology, IRCCS Istituto Giannina Gaslini, Genova, Italy; ^3^ Department of Experimental Medicine (DIMES), University of Genoa, Genoa, Italy

**Keywords:** nephrotic syndrome, rituximab, daratumumab, CD38, CD20, focal segmental glomerulosclerosis, minimal change disease of

## Abstract

Nephrotic syndrome affects about 2–7 per 100,000 children yearly and accounts for less than 15% of end stage kidney disease. Steroids still represent the cornerstone of therapy achieving remission in 75–90% of the cases The remaining part result as steroid resistant nephrotic syndrome, characterized by the elevated risk of developing end stage kidney disease and frequently presenting disease recurrence in case of kidney transplant. The pathogenesis of nephrotic syndrome is still far to be elucidated, however, efficacy of immune treatments provided the basis to suggest the involvement of the immune system in the pathogenesis of the disease. Based on these substrates, more immune drugs, further than steroids, were administered in steroid resistant nephrotic syndrome, such as antiproliferative and alkylating agents or calcineurin inhibitors. However, such treatments failed in inducing a sustained remission. In last two decades, the developments of monoclonal antibodies, including the anti-CD20 rituximab and inhibitor of B7-1 abatacept, represented a valid opportunity of treatment. However, also the effectiveness of biologics resulted limited. We here propose a new hypothesis-driven treatment based on the combining administration of rituximab with the anti-CD38 monoclonal antibody daratumumab (NCT05704400), sustained by the hypothesis to target the entire B-cells subtypes pool, including the long-lived plasmacells.

## Introduction

Glomerulonephritis accounts for less than 15% of kidney failure in patients less that 25 years old. Nephrotic Syndrome (NS) is a clinical entity characterized by fluid overload, dyslipidemia and hypoalbuminemia as consequence of massive proteinuria. NS is responsible for majority of glomerulonephritis and affects about 2–7 per 100,000 children aged below 18 years yearly (1, 2). In therms of histological lesions, NS may manifests as minimal change disease (MCD) in 80–90% of the patients or as focal segmental glomerulosclerosis (FSGS) in most of the remaining cases (3).

Pathogenesis of NS is still largely unknown. Historically, a T cell disorder resulting in the release of circulating factor(s) increasing the glomerular permeability to serum proteins was considered responsible of the disease. However, the identification of these permeability factor(s) still represents a challenge for nephrological community (4).

Despite lack of a clear disease pathogenesis, the use of immunosuppressive therapies was sustained by the immune abnormalities that characterized subjects with NS. Steroids still represent the cornerstone of therapy and induce remission in 80–90% of the cases (steroid-sensitive NS- SSNS) (5, 6). However, around half of subjects who initially responded to steroids develop recurrence after withdrawal and a chronic immunesuppressive treatment is requested to maintain remission, developing a steroid-dependent nephrotic syndrome (SDNS). Steroid-resistant nephrotic syndrome (SRNS) accounts for around 15% of overall cases (7). Children with SSNS, have a benign prognosis, while subjects with SRNS are characterized by an high risk of developing kidney failure. SRNS is also worsened by the elevated rate of recurrence after kidney transplantation (KT) in around half of cases (8). Moreover, long-term complications related to the chronic administration of steroids, are commonly reported. Steroid sparing agents, including mycophenolate mofetil (9, 10) or azathioprine (11), cyclosporine A or tacrolimus (CNI) (12) and others (13), are administered with the aim to limit overall steroid dose, but result ineffective in SRNS (14).

The recent development of new biologics paved the way for more selective and safer hypothesis-driven treatments. These therapies in the context of SRNS will be the focus of the present review.

## Monoclonal anti-CD20 antibodies

### Rituximab

Rituximab is a chimeric monoclonal antibody targeting the CD20 antigen. Rituximab administration was initially approved for the treatment of haematological and reumathological disease and results in na*i*ve and memory B cells depletion (15). Rituximab acts through the antibody-dependent and the complement-mediated cytotoxicity and through the induction of phagocytosis and apoptosis (16). In 2004, a young boy with SDNS secondary to MCD underwent proteinuria remission after receiving rituximab to treat supervened idiopathic thrombocytopenic purpura (17). After this initial case, other reports from patients receiving rituximab for post-transplant lymphoproliferative disorders (PTLD) indicated a potential benefit also on post- transplant FSGS recurrence (18, 19). Subsequently, there have been several randomised clinical trials reporting rituximab efficacy in SDNS (7).

On the other hand, the efficacy of rituximab in SRNS compared to common protocols, such as calcineurin inhibitors and plasma exchange, provided confounding results. Bagga et al. (20) treated with rituximab five SRNS children and and complete and partial remission were induced in three and two patients rispectively. After that, authors published a relevant case series reporting the treatment with rituximab of 33 SRNS paediatric patients: stable remission was achieved in around half of subjects at 12 months of follow-up (21).

In 2012, Magnasco et al. (22) proposed a randomised clinical study in 31 children with NS resistant to the combination of CNI and steroid. Authors compared the adding of two monthly infusions of rituximanb at the dose of 375mg/m^2^ in the treated group versus the maintenance of CNI and steroid in the control group: adding of rituximab did not result superior in inducing NS remission (**Table 1**).

**Table 1:** Main studies reporting biologics in SRNS.

Reference	Disease	N	Study design	Dose	CR (%)
Rituximab					
Bagga A, et al., 2007 (20)	SRNS	5	Case series	375mg/m^2^ (4 doses)	60
Gulati A, et al., 2010 (21)	SRNS	33	Multicentric Cohort Study	375mg/m^2^ (4 doses)	27
Magnasco A, et al., 2012 (22)	SRNS	31	RCT	375mg/m^2^ (2 doses) *vs* 375mg/m^2^ + Cyc	30
Garrouste C, et al., 2017 (23)	post-Tx recurrent FSGS	12	Case series	375mg/m^2^ (1-4 dose)	47
Lanaret C, et al., 2020 (24)	post-Tx recurrent FSGS	148	Multicentric Retrospective Study	375mg/m^2^ (1-4 dose)	47
Ofatumumab					
Basu B, et al., 2014 (25)	multidrug resistant NS	5	Case series	300mg/m^2^ followed by 5 weekly infusions (2g/m^2^)	80
Bonanni A (26), et al., 2015	multidrug resistant NS	4	Case series	375-700 mg/m^2^ (1 dose)	25
Wang CS, et al, 2017 (27)	SRNS	4	Case series	300mg/m^2^ followed by 5 weekly infusions (2g/m^2^)	75
Ravani P, et al, 2020 (28)	multidrug resistant NS	7	RCT	1,500mg/m^2^ (1 dose)	0
Bernard J, et al, 2020 (29)	post-Tx recurrent FSGS	6	Case series	300mg/m^2^ followed by 5 weekly infusions (2g/m^2^)	0
Abatacept					
Yu CC, et al., 2013 (30)	FSGS	5	Case series	10mg/kg (1-3 doses)	100
Garin EH, et al., 2015 (31)	MCD/ FSGS	5	Case series	10 mg/kg (2 doses)	0
Delvlille M, et al., 2016 (32)	post-Tx recurrent FSGS	9	Case series	10 mg/kg (1-13 doses)	0
Burke GW, et al, 2023 (33)	post-Tx recurrent FSGS	12	Case series	10 mg/kg (1-13 doses)	60
Adalimumab					
Joy MS, et al., 2010 (34)	FSGS	10	Phase I clinical trial	24mg/m^2^ (9 doses)	N/A
Anakinra					
Angeletti A, et al., 2022 (35)	SRNS/post-Tx recurrent FSGS	3	Case series	200mg/daily	33

Data are presented as follow-up period for all the patients or as median (range); CR, complete remission; N/A, not available, RTX, rituximab; RCT, randomized controlled trial; SRNS, steroid resistant nephrotic syndrome; Tx, kidney transplantation.

Overall, observational studies suggested a complete remission rate of about 30% of cases treated with rituximab (36), but the limited controlled randomised studies did not support the superiority of rituximab compared to other treatments.

Similar results were described in the context of NS recurrence after KT. NS recurrence is reported in 35-60% of cases and represents a major risk factor for graft lost (37). Rituximab and plasmapheresis represented the corner-stones of treatment in NS recurrence, but remission was generally described in 40-55% of cases and directly correlates with better graft survival (38).

Rituximab administration in post KT recurrence was largely reported, but literature mostly consists of case reports and case series (39–42). Remission rate of subjects treated with rituximab for NS recurrence ranged from 50% to 100%. While other reports showed total failure of rituximab in inducing remission (43–45). Moreover, the dose of rituximab used to treat NS recurrence is variable according to the centers and represents a further confounding factor in interpreting previous reports: dose administered ranges from single infusion at low-dose of 100 mg to 2 monthly infusions of 1 gr, according to the centers’ protocols (46).

In 2017, Garrouste et al. (23), in a retrospective observational study, analysed the administration of rituximab in 19 patients with recurrent NS post KT. They reported 9/19 and 3/19 complete and partial remission respectively. Moreover, responsiveness to treatment correlated with a better renal function at 5 years of follow up. Of note, 14 patients reported severe infections in the first year of follow up. More recently, the same authors investigated the efficacy of rituximab in a large retrospective multicenter study on 148 KT recipients with FSGS recurrence, maintaining plasmapheresis as cornerstone of treatment (24). Overall, complete and partial remission were achieved in 47% and in 33%of patients, respectively (**Table 1**). Of interest, the patients here reported where divided in two groups according to the time of administration of rituximab: the administration of rituximab for recurrence prevention at the day of transplantation did not correlate with higher incidence of remission or a decreased frequency of second relapse when compared to the administration of rituximab only in case of relapse.

Uffing et al. (38), in TANGO project (47), retrospectively screened for FSGS 11,742 KT recipients and reported that 176 had a diagnosis of native FSGS and 57 (32%) had recurrence after KT. Rituximab and plasmapheresis represented treatments of choice in 81% of cases, and overall, partial or complete remission was reported in 57% of patients, correlating with better survival of KT.

Regarding our experience, we revised the outcomes of 25 non-genetic SRNS who received KT at Gaslini Children’s Institute, Genoa, Italy, between 1988 and July 31, 2022. We here reported a recurrence rate of 45% (11/25). Patients were treated with plasmapheresis or plasmapheresis and rituximab, achieving a remission rate of 60% of recurrent cases. We did not report differences among who received or not rituxumab.

A meta-analysis recently evaluated the evidence of combining plasmapheresis with rituximab therapy. Authors considered eight observational studies including both adults and pediatrics age groups, and they found that the remission rate was 72.7%, 41% with complete and 32% with partial remission respectively (48).

Summarising, although there are no randomized control trials on the effectiveness of rituximab in treating post KT recurrent NS, the effectiveness of both rituximab and plasmapheresis is largely supported.

### Safety of rituximab

Rituximab is generally well tolerated. The infusion related reactions represent the most common complications and occur in 5–10% of the cases. In our recent clinical trial comparing efficacy and the safety of rituximab vs. ofatumumab, we demonstrated that infusion related reaction may be avoided with slow infusion and with pre-treatment based of steroid, antihistaminic, paracetamol and salbutamol.

In NS, serious adverse events in children receiving rituximab were reported in limited case reports, such as pulmonary fibrosis (49) fulminant myocarditis (50). Different complications, including leukoencephalopathy (51), do not seem related to rituximab, considering that also the administration of other immunosuppressive agents correlated with the JC virus reactivation (52). Moreover, previous findings largelly reported that chimeric nature of rituximab may be responsible of the development of anti-rituximab antibodies (53, 54). As consequence, anti-rituximab antibodies may limit the efficacy of subsequent infusions and may increase the infusion related reactions (55). We recently reported that, in our patients with SDNS, the development of anti-rituximab antibodies is limited and does not affect the efficacy of subsequent infusions, probably due to the low dose administered (single infusion 375mg/m^2^) compared to other protocols (56).

In a recent retrospective study, Bagga et al. (57) investigated the outcome of 250 patients with SDNS receiving two or more courses of rituximab. Authors reported that hypogammaglobulinemia was observed in around 35% of treated subjects. However that hypogammaglobulinemia may persist several months after the last rituximab infusion is commonly reported, but if the hypogammaglobulinemia would be related to a major risk of severe infections is still to be completely defined (58).

### Ofatumumab

Ofatumumab is a fully human anti-CD20 monoclonal antibody. At development of the drug, ofatumumab presented different *in vitro* characteristics compared to rituximab, such as more extended binding site with consequent more affinity to the CD20 antigen and an higher efficient complement-dependent cytotoxicity (59). Such *in vitro* findigs raised the curiosity on the clinical administration with the aim to investigate possible superior clinical benefits.

In neprhrological context, ofatumumab was firstly administered in five children with SRNS

Not responding to rituximab at the dose of 300 mg/1.73m^2^ followed by five weekly infu- sions (2 g/1.73m^2^ *per* infusion). At 12 months of follow up, complete and partial remission were achieved in three and two subjects respectively (25). Such satisfactory results were not reported by further experiences (26). Vivarelli et al. (60) described the administration of ofatumumab in two cases of steroid and rituximab resistant NS, responding to a single dose. Wang et al. (27) treated with ofatumumab five patients with NS at dose of 300 mg/1.73m^2^ for the first week, followed by four or five weekly infusions of 2 g/1.73m^2^. One failed the treatment for severe infusion reactions. Three achieved complete remission, and one partial remission. In a more recent randomized controlled trial in children with multi-drug NS (MRNS), ofatumumab (single infusion at 1,5 g/1.73m^2^) was compared with placebo and failed to induce remission (28). Overall, ofatumumab induced NS remission in around 40% of treated children for SRNS (29) (**Table 1**).

Based on single case reports and small series (61–65), ofatumumab was also proposed in the treatment of NS recurrence after KT. However, in the largest case series involving 6 children resistant to previous treatments, ofatumumab did not induce complete remission, partial remission was achieved in half of cases and no response at all in the remaining cases.

## Other biologics

### Abatacept

*A*ntigen-presenting cells expressed on the surface a costimulatory ligand named B7-1 (CD80), fundamental for the binding with the T- cell receptors CD28 and Abatacept is an inhibitor of the B7-1 (66). Previous findings described the B7 expression on podocytes surface of subjects affect by proteinuric disease, such as lupus nephritis (67), membranous nephropathy (30) and diabetic nephropathy (68, 69). Therefore, such reports raised the hypothesis that B7 blockade may result in a podocyte-protective effect with consequent reduction of proteinuria. In a case report study, partial or complete remission was reported in 4 KT recipients with rituximab-resistant recurrent FSGS and treated with Abatacept and in one SRNS. Muhlbacher et al. (70) described a 19-years old boy with recurrent NS after KT and resistant to rituximab and plasmapheresis, who responded to abatacept and was mentained in remission with subsequent belatacept-based immunosuppression with a follow-up of over 4 years (**Table 1**). Other studies reported confunding and not definitive results (31, 32).

More recently, Burke et al. (33) proposed the administration of abatacept, based on the B7-1 podocytes expression at kidney biopsy, in 12 subjects (median age 12 years old) with NS recurrence after KT and resistant to conventional treatments with plasmapheresis and rituximab. Nine subjects responded to treatment, of whom 7/9 had KT biopsy positive for B7-1, while 2/9 were without biopsy. Of note, of the 3 patients not responding to abatacept, one had KT biopsy positive for B7-1 (**Table 1**). Based on these results, authors suggested that B7-1 podocytes staining may identify subject who can benefit of abatacept.

These evidence may support the need for confirmatory studies on podocyte B7-1 expression and randomized clinical trial to asses the response to abatacept in proteinuric kidney disease.

### Adalimumab

Adalimumab is a human monoclonal antibody anti-TNFa. It is approved for the rheumatoid and gastric disease, such as children affected by juvenile idiopathic arthritis, psoriasis Crohn’s disease and arthritis (71). TNFa was reported elevated in urine of subjects affected by FSGS (72, 73). Moreover, *in vivo* studies demonstrated that TNFa may induce endothelial cell injury and increased glomerular permeability (74). Therefore, based on these previous findings, adalimumab was supposed to be a promising treatment for proteinuric disease. In a Phase 1 clinical trial, adalimumab was effective in decreasing proteinuria in 4/10 subjects with FSGS (34)(**Table 1**).However, further clinical trials are missing and current data still do not support adalimumab use in FSGS.

### Anakinra

In animal models of FSGS, regulation of C3 convertase in podocyte by decay-accelerating factor (CD55) is fundamental to enhance proteinuria and glomerular sclerosis. Recent studies showed that C3a/C3aR ligations on podocytes induce the podocytes to release active IL-1β that through the ligand with type 1 interleukin receptor (IL-1R1), with an autocrine loop, lead to the actin cytoskeleton rearrangement and podocyte loss. The loss of podocytes may be avoided preventing the binding IL-1β/IL-1R1, suggesting therefore a causal link (75, 76).

Since 1993, Anakinra, the receptor antagonist that blocks the activity of IL-1 binding the IL-1R1, represents the treatment of choice in several rheumatic disease (77). Of note, several previous studies reported the use of Anakinra in patients with familial Mediterranean fever and associated glomerular AA amyloidosis resulting with proteinuria: the administration of Anakinra remarkably reduced the amount of proteinuria (78). Such findings may be partially explained by the previous described mechanism correlating IL-1β production and the subsequent IL-1R signaling to the complement cascade during proteinuric disease.

Based on these findings, we recently administered Anakinra in 2 patients with multidrug-dependent/-resistant NS and 1 KT patient with FSGS recurrence. We induced a complete and two partial remission respectively. Therefore, our small case reports supported the hypothesis that IL-1R1 blockers may prevent the complement-mediated podocyte cytoskeleton rearrangement in glomerular diseases (35) (**Table 1**).

## New hypothesis-driven treatment, combining rituximab and daratumumab

B cells play a role in the pathogenesis of NS, as reflected by the success of monoclonal anti-CD20 antibodies in the treatment of SSNS. However, as previously reported, the effect of rituximab in complicated NS is limited (22). On the other hand, B-cells exist as different subsets reflecting the different maturation stages in the peripheral blood. Rituximab targets the majority, but not the totality, of the B-cells subtypes. Indeed, unlike short-lived plasmablasts, long-lived plasmacells are unresponsive to anti-CD20 immunosuppressive treatments. Plasmacells highly express CD38, a glycoprotein with ectoenzymatic functions. CD38 is a myeloid antigen present both on the cell membrane and in the intracellular compartment of the cell. Several physiological and pathological conditions, such as the early phases of the immune response or the inflammatory disease (i.e. systemic lupus erythematosus and rheumatoid arthritis) may lead to the increased expression of CD38. It is highly expressed in the B-cells starting from the germinal center. Both T and B-cells upregulate CD38 expression upon activation, while NK cells seem to express it on a constant level. Specific cancer cells, such as multiple myeloma cells, express high levels of CD38 glycoprotein. Therefore, the anti-CD38 monoclonal antibody daratumumab is administered in multiple myeloma and it induces a substantial decrease of the bone marrow malignant plasma cells.

The relevance of targeting long-lived plasmacells in proteinuric disease, such as lupus nephritis (79, 80) and membranous neophropathy (81), have already been reported in previous clinical reports. Unlike to lupus nephritis and membranous neophropathy, that represent clear examples of autoantibodies mediated disease, the role of long-lived plasmacells in SRNS is not completely explored. However, despite the absence of a clear pathological mechanism for SRNS, the increased glomerular permeability to protein is also sustained by an imbalance between conventional and regulatory Tcells. Of interest, daratumumab may directly affect T-cells subtypes: Ostendorfet al. (79), described two cases of Systemic Lupus Eritematosus (one with lupus nephritis), unresponsive to standard immunosuppression, with substantial clinical response to daratumumab (82). They investigated the effect of daratumumab on T-cell function through single-cell transcriptome analysis and reported the alterations of the transcriptional profile of CD4+ and CD8+ T cells, as main consequence of treatment with daratumumab.

On this basis, it seems reasonable to consider the association of two drugs, one targeting CD20 and one targeting plasmacells. In recent clinical study, the humanized anti-CD20 antibody obinutuzumab (single dose 1 g/1.73m^2^) was combined with the anti-CD38 monoclonal antibody daratumumab (single dose 16mg/kg) in 14 subjects with SDNS/frequent-relapsing NS that relapsed after previous treatments with rituximab. As main results, authors reported that the association of obinutuzumab and daratumumab induced a remission period longer than previous treatment with only rituximab in the same subjects (83). However, the achieved remission period resulted quite similar to other randomised clinical trials testing the only administration of monoclonal antibody anti-CD20. Therefore, despite the significative results and the safety of the treatment proposed by Dossier et al., we do not consider patients affected by SDNS the right target to be treated by the association of anti-CD20 and anti-CD38 antibodies. On the other hand, the same group described the efficacy of the combined treatment with obinutuzumab and daratumumab in a young subject with post-transplantation SRNS recurrence and resistant to standard treatments (84).

**Figure 1:**
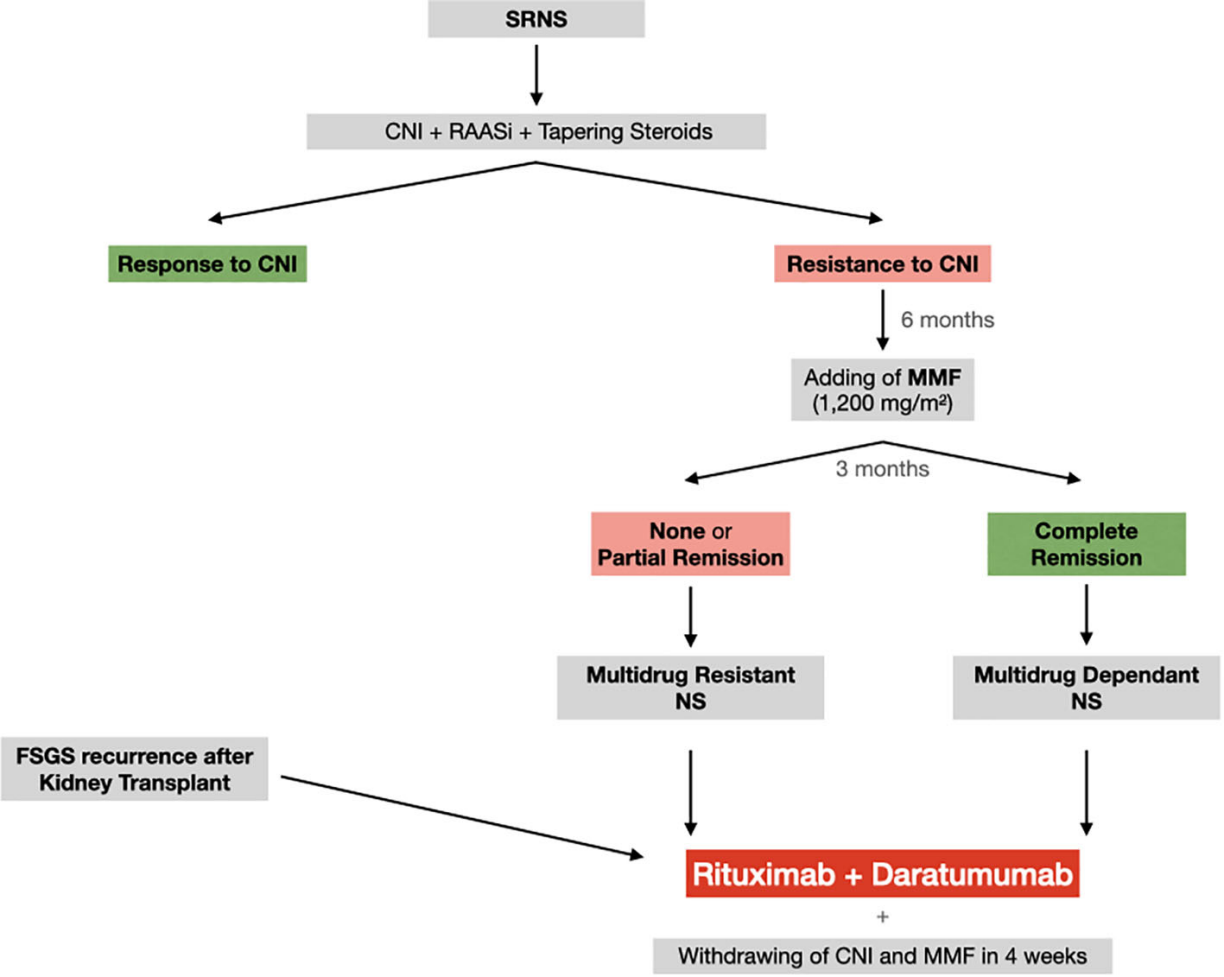
Flow-chart of the study. CNI, calcineurin inhibitors; FSGS, focal segmental glomerulosclerosis; MMF, mycophenolate mofetil; RAASi, renin angiotensin aldosteron inhibitors.

Therefore, the preliminary positive results of their combination may open new opportunities in the treatment of NS and supports the need for new studies in those patients who require more than one drug to maintain remission. Therefore, we recently proposed a phase 2 proof-of-concept study (NCT05704400, DUAL-1) for testing the superiority of rituximab (single dose 375mg/m^2^) plus daratumumab (single dose16mg/kg) in maintaining drug-free disease remission in children and young adults (3-24 years) affected by MDNS, SRNS and post transplant NS recurrence. More in details, MDNS is defined by the need of at least 2 of the oral drugs, including steroid, mycophenolate mofetil and calcineurin inhibitors. Resistance is defined by lack of NS response to double therapy consisted of steroid, mycophenolate mofetil and calcineurin inhibitors. After the administration of the combined treatment with rituximab and daratumumab, ongoing immunosuppressive treatment will be shortly withdrawn. In **Figure 1** we reported the flow-chart of the proposed study.

As main aim, we will evaluate the time-free remission in MDNS. On the other hand, in subjects affected by SRNS and post transplant NS recurrence, the decrease of proteinuria will represent the first endpoint. All patients will be enrolled after informed consent is obtained from patients and parents.

## Conclusions

SRNS represent a condition interesting a small number of people compared to the general population, but with devastating consequences in patients affected. SRNS is characterized by a fast progression of kidney failure and it is worsened by an high risk of recurrence after KT. Despite the relevant impact in nephrological clinical practice, SRNS still represent a challenge in terms of pathological mechanisms and effective treatment. Acute and chronic adverse events associated with chronic use of steroids and immunosuppressive drugs sustained the need for alternative therapeutical strategies. Despite initial expectations, rituximab have not represented a valid oportunity for subjects affected by SRNS.

We here propose a new hypothesis-driven treatment based on the combining administration of anti-CD20 rituximab with the anti-CD38 monoclonal antibody daratumumab, sustained by the hypothesis to target the entire B-cells subtypes pool.

## Author contributions

AA, MB, EP, SS, XK, GC, FL, EV and GG contributed to conception and writing of the work. AA and GG revising it critically. All the authors provide approval for publication of the content.
